# 

**Published:** 1895-04-27

**Authors:** 


**Notices of Births, Deaths, and Marriages** are inserted in this column at a charge of 2s. 6d. for an announcement not exceeding four lines.

**Notice to Advertisers and Subscribers.**

All Advertisements, Orders for Copies of The Hospital and Business Communications should be addressed to **The Manager, NOT TO THE EDITOR,** The Hospital, 428, Strand, London, W.C.

The Cost of Subscription, post free, is as follows:—Per week, 2½d.; per quarter, 2s. 3d.; per half-year, 5s. 3d.; per annum, 10s. 6d. Foreign:—Per Annum, 15s. 2d.; payable, in all cases, in advance.

**NOTICE TO CORRESPONDENTS.**

All MS., Letters, Books for Review, and other matters intended for the Editor should be addressed The Editor, The Lodge, Porchester Square, London, W.

The Editor cannot undertake to return rejected MS., even when accompanied by stamped directed envelope.

**"Burdett's Hospital Annual," 1895,**

Ready Immediately.

Sixth year of publication. About 1000 pp. Scarlet cloth, gilt lettered. Price 5s. A most complete guide to British, Colonial, Indian, and American Hospitals, Dispensaries, Nursing and Convalescent Institutions, and Asylums. A few copies of the "Annual" for 1894 may still be obtained on application to the Publishers, The Scientific Press (Limited), 428, Strand, London, W.C.

**NOTICE.—Vol. XVII. of "The Hospital" (October, 1894, to April, 1895), in handsome cloth binding, is now on sale at the Office of this Journal. Price 6s. Cases for binding the Numbers contained in this Volume, 1s. 6d. Index to Vol. XVII., 2½d., post free.**

### SCRAPS AND GLEANINGS.

The site has been chosen for the new fever hospital at Camelon, Stirling.

The Town Council of Brighton have resolved to spend over £20,000 on a new sanatorium.

The committee of the Durham County Hospital Ball have handed over £38 this year to the treasurer of the hospital.

The Armourers and Brasiers' Company has presented 100 guineas to the special appeal fund of St. Thomas's Hospital.

In connection with the Bristol Eye Hospital, a special fund has been started to help the poorest patients to buy spectacles.

An attractive ballad concert was held last week at the Shoreditch Hall in aid of the North-Eastern Hospital for Children in Hackney Road.

A donation of £500 has been given to the Cottage Hospital at Horsham by Mr. Harben, M.P., as a contribution to the endowment fund.

The Marquis Camden will open a bazaar at the Athenæum, Kentish Town, in aid of the North-West London Hospital, Kentish Town, on Monday, May 13th, at three o'clock.

The amalgamation of the Nottingham Samaritan Hospital for Women with the Women's Hospital in Castle Gate was suggested at the recent annual meeting of the former institution.

A bazaar in aid of the furnishing fund of the Derbyshire Royal Infirmary was opened by the Duchess of Teck at Derby on Easter Tuesday, to continue during the four succeeding days.

The festival dinner of the East London Hospital for Children will be held on May 29th, at the Hall of the Worshipful Company of Leathersellers. The Earl of Errol has consented to preside.

A joint isolation hospital for the Malvern district is under discussion, the majority of Malvern doctors having expressed their opinion that such a hospital is necessary for the welfare of the district.

With the view of erecting a properly equipped dental hospital at Glasgow, the directors have had plans and estimates prepared. It seems that a suitable building could be procured for £3,000.

A cheque for £104 3s. 1d. has been forwarded to the Metropolitan Hospital, Kingsland Road, by the Shoreditch Concert Committee, being the proceeds of a concert held in the Town Hall in January.

The first annual meeting of the Glasgow Hospital Sunday Fund was held last month. The amount collected from the churches and Sunday-schools during the past year was £5,208 13s. 11d. December 1st, 1895, is the date fixed for the next collection.

One of the attractions of the forthcoming bazaar at Ballsbridge, in aid of one of the Dublin hospitals, is to be an exhibition by Professor Fitzgerald of an aerial machine, invented by the German scientist, Herr Libienthal.

A meeting was held last month of the Managing Committee and of the Board of Governors of the Royal Hospital for Incurables at Dublin. The banking account was stated to be overdrawn to the amount of £1,381 14s. 4d.

The second annual church parade and demonstration by members of the South-East London Friendly Trade and Temperance Societies took place recently in aid of the funds of St. Thomas's Hospital and the South Lambeth Dispensary.

A special meeting of the Council of the Charity Organization Society has been held to discuss the advisability of discouraging street collection by children on behalf of hospitals and other charities, it having been stated that money collected in this way has in many instances been misappropriated by the children.

Professor Sir William Turner has been presented by his colleagues and old pupils with his portrait (painted by Sir George Reid, President of the Royal Scottish Academy), in recognition of his services to science, and more especially to the University of Edinburgh. There was a large gathering for the occasion in the anatomy lecture theatre, Sir James A. Russell being in the chair.

At the recent annual meeting at the Mansion House in connection with the Hospital Saturday Fund it was stated in the report that the amount allotted during the past year was £17,608, and the Surgical Appliances Committee had expended £1,768 on the purchase of surgical appliances. A resolution was agreed to recommending the use of collection boxes in workshops for weekly contributions to the Fund.

Messrs. J. S. Fry and Sons, of chocolate fame, have just opened a new factory in Bristol, and celebrated the occasion by entertainments on a very large scale to all their employés and others. Altogether some 3,500 guests were invited to a sumptuous tea at the Colston Hall, the festivities extending over two evenings. Many members of the great firm were present, Mr. J. Storrs Fry presiding, and the official and medical staffs of most of the Bristol hospitals were represented amongst the visitors.

### Books Received.

J. B. Lippincott Co., Philadelphia.
"Suggestions to Hospital Visitors." Billings and Hurd.
T. Fisher Unwin.
"The Female Offender." Lombroso.
The St. Bride's Press.
"Drainage Work and Sanitary Fittings." William H. Maxwell.
Fanin and Co., Dublin.
"Transactions of the Royal Academy of Medicine in Ireland."
S. W. Partridge and Co.
"Benjamin Lownson." By His Daughter.

**Periodicals and Pamphlets.**—Westminster Review, Public Health, Clinical Sketches, Science News, Charity Organisation Review, The Modern Trained Nurse, Electrical Review, American Chemical Journal, The Sun, Strand Magazine, Strand Musical Magazine, Picture Magazine, The London Home Monthly, Dental Record, Guy's Hospital Gazette, Clinical Sketches, Revue Generale des Sciences, The Planter.

### The Student of Medicine.

### APPOINTMENTS.

H. Bamber, M.B., C.M.Glasg., appointed Medical Officer for the Tynemouth District of the Tynemouth Union. G. A. Barrow, M.R.C.S.Eng., L.R.C.P.Lond., appointed Junior House Surgeon to the Bolton Infirmary for a period of twelve months. W. Owen Evans, L.R.C.P., L.R.C.S.Edin., appointed Medical Officer for the Hawarden District. H. J. Finch, M.R.C.S.Eng., L.R.C.P.Lond., appointed Assistant House Surgeon to the Royal Surrey County Hospital, Guildford. Frank Gowers Gardner, M.R.C.S., L.S.A., appointed Surgeon to the Warwick Cottage Hospital and Dispensary. William Guy, F.R.C.S., L.R.C.P., L.D.S.Edin., appointed Dental Surgeon to the Royal Infirmary, Edinburgh. T. E. Harper, L.R.C.P., Assistant Medical Officer, Peckham House, S.E., appointed a Junior Assistant Medical Officer of Holloway Sanatorium Hospital for the Insane, Virginia Water. A. Conning Hartley, M.D.Edin., F.R.C.S., appointed Medical Officer for the Bedford and Kempston District of the Bedford Union. J. McMurray, M.D., M.Ch., appointed Honorary Surgeon to the Bootle Borough Hospital. W. H. Mardlow, appointed House Surgeon to the Taunton and Somerset Hospital. William Robert Martine, M.B., C.M.Edin., appointed Resident Surgeon to the Edinburgh Royal Infirmary. J. A. Morris, L.R.C.P., L.R.C.S.I., appointed Medical Officer for the Kilkenny Workhouse. R. L. S. Nuthall, L.R.C.P.Lond., Assistant Medical Officer of Hanwell Asylum, appointed Assistant Medical Officer with charge of the male side of Holloway Sanatorium Hospital for the Insane, Virginia Water. J. Porter Parkinson, M.D., M.R.C.P.Lond., F.R.C.S. Eng., appointed Medical Registrar to the Westminster Hospital. Edward B. Roberts, L.R.C.P. and S.Edin., appointed Medical Officer of Health for the Hawarden District Council and District Medical Officer for the Buckley District. E. H. Squire, M.R.C.S.Eng., L.S.A., appointed Medical Officer for the Second District of the Lexden and Winstree Union. Mr. Turner, appointed Medical Officer and Public Vaccinator for the No. 4 District of the Chipping Norton Union.

### VACANCIES.

#### Resident Medical Officers.

General Hospital, Birmingham.—Assistant House Surgeon, must possess a surgical qualification. Appointment for six months. No salary, but residence, board, and washing provided. Applications to Howard J. Collins, House Governor, by April 27th.

Hospital for Sick Children, Great Ormond Street, Bloomsbury, W.C.—Resident House Physician; also House Surgeon. Appointment for six months. Salaries £20, with board and residence in the hospital. Unmarried, and possess a legal qualification to practice. Applications and testimonials to Adrian Hope, Secretary, before Tuesday, April 30th.

Kent County Lunatic Asylum, Barming Heath, near Maidstone.—Fourth Assistant Medical Officer and Pathologist; unmarried. Salary £175 per annum, rising £5 annually, with furnished quarters, attendance, coal, gas, produce, milk, and washing. Appointment for two years in the first instance. Applications to Francis R. Howlett, Clerk to the Sub-committee of Visitors, 9, King Street, Maidstone, by May 1st.

Weston-super-Mare Hospital and Dispensary.—Medical Officer to the Provident Dispensary; must possess a recognised medical and surgical qualification; must be registered under the Medical Act. Salary £80 per annum, with board, lodging, and washing. Applications and testimonials to the Honorary Secretary before April 30th.

### THE STUDENT OF MEDICINE—*continued.*

### Non-Resident Medical Officers.

Charing Cross Hospital.—Curator and Pathologist. Salary £100 per annum. Applications to Arthur E. Reade, Secretary, by April 29th.

Denbighshire Infirmary and General Dispensary, Denbigh.—Honorary Medical Officer, doubly qualified. Applications to the Chairman of the Committee of Management by May 14th.

Grocers' Company.—Three Medical Research Scholarships, each of the value of £250. Appointments annually. Applications to the Clerk of the Company, Grocers' Hall, Princes Street, E.C., by the end of April.

Middlesex Hospital, W.—Assistant Surgeon; must be Fellow (or have passed the qualifying examination for the Fellowship) of the Royal College of Surgeons of England. Applications to F. Clare Melhado, Secretary Superintendent, by May 1st.

National Hospital for the Paralysed and Epileptic, Albany Memorial, Queen Square, W.C.—Registrar. Honorarium of 50 guineas is attached to the post. Appointment tenable for two years, and for a longer period subject to re-election. Applications to B. Burford Rawlings, Secretary and General Director, by May 3rd.

University of Glasgow.—Two Examiners for Degrees in Medicine to examine in Clinical Medicine and Clinical Surgery respectively. Appointment to last till December 31st, 1898, at the rate of £50 annually. Applications to Alan E. Clapperton, Secretary of the Glasgow University Court, 91, West Regent Street, Glasgow, by May 1st.

### UNIVERSITIES AND COLLEGES.

EXAMINING BOARD IN ENGLAND BY THE ROYAL COLLEGES OF PHYSICIANS AND SURGEONS.

The following gentlemen passed the Second Examination of the Board in the subjects indicated. Monday, April 1st:—

Anatomy and Physiology.—I. Taylor, student of Yorkshire College, Leeds; R. W. Pearson, H. H. Robinson, J. Bennet, and J. Wood, of Owens College, Manchester; J. Black, of Owens College, Manchester, and Firth College, Sheffield; J. Gardner and N. Milner, of Firth College, Sheffield; F. Barnes, F. N. Deakin, W. H. Coltart, J. G. C. Taunton, J. Bradley, F. Pope, and J. A. N. Longley, of Mason College, Birmingham; A. W. Tuxford, of St. Mary's Hospital; W. R. Battye, of University Colleges, Bristol and London; and J. W. Hughes, of Cambridge University.

Anatomy only.—H. Spinks, J. Wallwork, and H. Blakemore, of Owens College, Manchester; F. C. Morgan and C. M. Mitchell, of University College, Bristol.

Physiology only.—G. Renshaw and H. L. Laidman, of Owens College, Manchester; and O. T. A. Phillips, of University College, Cardiff.

Twelve gentlemen were referred in both subjects, four in Anatomy only, and five in Physiology only.

Tuesday, April 2nd.

Anatomy and Physiology.—W. E. Jones, student of Owens College, Manchester; W. E. B. Roberts, of Mason College, Birmingham; E. C. Sawdy and E. S. Graham, of St. Mary's Hospital; E. V. Foss and R. F. Moorshead, of University College, Bristol; G. L. Bates and W. J. May, of Charing Cross Hospital; R. W. C. Pierce, W. H. Tucker, and H. E. Hewitt, of St Thomas's Hospital; G. C. Marrack, of St. Bartholomew's Hospital: H. J. Greant, of Westminster Hospital; L. E. C. Handson, of Guy's Hospital; T. Jones, of London Hospital; A. Roman, of Strassburg, Tubingen, and Keil Universities; and H. A. Bruce, of Toronto University and University College, London.

Anatomy only.—E. S. Crispin, of King's College, London; J. L. Payne, of Guy's Hospital; A. J. Andrew, of St. Bartholomew's Hospital; P. C. Maitland, of Middlesex Hospital; and J. E. H. Parsons, of Cambridge University and Guy's Hospital.

Physiology only.—J. M A. Manning, of St. George's Hospital; D. Ackland, of Charing Cross Hospital; and A. R. O'Flahertie, of London Hospital and Mr. Cooke's School of Anatomy and Physiology. Ten gentlemen were referred in both subjects, five in Anatomy and six in Physiology only.

### Medical and Administrative Appointments.

**THE HOSPITAL FOR SICK CHILDREN,**
GREAT ORMOND STREET, BLOOMSBURY, W.C.

